# Plasma Exosomal hsa_circ_0015286 as a Potential Diagnostic and Prognostic Biomarker for Gastric Cancer

**DOI:** 10.3389/pore.2022.1610446

**Published:** 2022-06-09

**Authors:** Peiming Zheng, Huijie Gao, Xuanhu Xie, Peipei Lu

**Affiliations:** ^1^ Department of Clinical Laboratory, Henan Provincial People’s Hospital, People’s Hospital of Zhengzhou University, People’s Hospital of Henan University, Zhengzhou, China; ^2^ Department of Oncology, The First Affiliated Hospital of Henan University, Kaifeng, China

**Keywords:** biomarker, gastric cancer, diagnosis, CircRNA, exosomes

## Abstract

Circular RNA (circRNA) is stable and abundant in exosomes as a potential biomarker for the diagnosis and prognosis of tumor. In this study, cancer specific exosomal circRNAs were identified through circRNA microarray, and 58 circRNAs were significantly upregulated in cancer cells derived exosomes. Then 60 patients with newly diagnosed gastric cancer (GC), 30 chronic gastritis patients and 30 healthy subjects were enrolled for further clinical validation. We detected that hsa_circ_0015286 was remarkably highly expressed in GC tissue, plasma and cancer cells compared with normal controls. Results of ROC curve analysis showed that the area under curve (AUC) of hsa_circ_0015286, CEA and CA 19-9 was 0.778, 0.673, and 0.665, respectively. The combined detection of three indicators had the highest AUC (0.843). Exosomal hsa_circ_0015286 expression was closely associated with tumor size, TNM stage and lymph node metastasis. The expression level of exosomal hsa_circ_0015286 in GC patients decreased significantly after surgery. Overall survival of patients with low hsa_circ_0015286 expression was longer than those with high expression. Our data demonstrated that exosomal hsa_circ_0015286 might be a promising noninvasive biomarker for the diagnosis and prognosis evaluation of GC.

## Introduction

Gastric cancer (GC) is the fifth most common malignant tumor and the third leading cause of cancer-related death worldwide, with over one million new cases in 2020 and an estimated 769,000 deaths globally [1]. GC remains the second most frequent cancer in China, and most patients are diagnosed at later stage with a lower five-year survival rate due to the finite early diagnosis [2, 3]. Therefore, it is urgent to find novel non-invasive biomarkers for diagnosing and prognosis monitoring of patients with GC.

Exosomes, one type of extracellular vesicles (EVs) with a diameter range of 30–150 nm, are secreted by almost all cells. More importantly, exosomes are widely existed in blood, urine, cerebrospinal fluid, saliva, and other common body fluids, comprising a variety of lipids, proteins, DNAs and noncoding RNAs (ncRNAs) derived from the original cells [4, 5]. Moreover, due to the protection of the lipid bilayer structure of exosomes, these disease-specific DNAs and RNAs in exosomes are stable and ideal biomarkers for diagnosis and prognosis monitoring of most diseases [6, 7]. Circular RNA (circRNA) is a particular endogenous RNA with a covalently closed loop that is distinguished from the traditional linear RNA [8]. At present, circRNAs have been found to be stable and enriched in exosomes [9]. Recent studies have confirmed that circulating exosomal circRNAs can reflect the malignant characteristics and progression of cancer, implying their significant potential as novel diagnostic and prognostic biomarker for cancer [10, 11]. Therefore, we assumed that GC cells in the tumor microenvironment are able to produce and deliver exosomes, and the detection of plasma exosomal circRNAs might supply a noninvasive and convenient approach for the diagnosis and prognosis evaluation of GC.

In this study, based on the result of circRNA microarray in cancer cell-derived exosomes, hsa_circ_0015286 was selected and proved to be highly expressed in plasma and tissue of GC patients. The diagnostic and prognostic values were evaluated using receiver operating characteristic (ROC) curve and Kaplan-Meier survival curve. Then, the association between the expression level of plasma exosomal hsa_circ_0015286 and the clinicopathological parameters of GC patients was further analyzed. Our data illustrated the potential of exosomal hsa_circ_0015286 as a noninvasive biomarker for the diagnosis and prognosis evaluation of GC.

## Material and Methods

### Cell Culture

Human gastric epithelial cell line (GES-1) and four cancer cell lines (BGC823, SGC7901, MGC803, AGS) were purchased from the Chinese Academy of Sciences Cell Bank of Type Culture Collection. Three of the four cancer cell lines we commonly used (BGC823, SGC7901 and MGC803) are actually HeLa derivatives. Cells were cultured in high-glucose DMEM (HyClone) medium at 37°C with 5% CO_2_, all media were supplemented with 100 μg/ml streptomycin, 100 U/ml penicillin and 10% exosome-depleted Foetal Bovine Serum (FBS, Gibco). For exosome extraction, culture medium (CM) was collected and centrifuged at 2,000 g for 10 min, then followed by 12,000 g for 10 min at 4°C. The supernatant was transferred and stored at −80°C.

### Patients

Sixty newly diagnosed GC patients admitted to the First Affiliated Hospital of Henan University from October 2018 to December 2019 were selected. The average age of the GC patients was 63.7 (range from 38 to 81 years old), with 32 males and 28 females. The inclusion criteria were as follows: 1) all subjects were confirmed by gastroscopy or histopathology; 2) no any chemotherapy or surgical treatment before; 3) with complete clinicopathological features. The exclusion criteria were as follows: 1) together with heart, kidney, and liver dysfunction; 2) together with other cancer. Tumor clinical stages and histological grades were assessed according to the 8th AJCC/TNM staging system and National Comprehensive Cancer Network clinical practice guideline of oncology (V.1.2012), including 10 stage I patients, 17 stage II patients, 19 stage III patients and 14 stage IV patients. Lymph node metastasis was identified that metastasis to intraabdominal lymph nodes, including hepatoduodenal, retropancreatic, mesenteric, and para-aortic.

Besides, thirty of the above sixty patients received surgical treatment, 30 paired GC tissues and paracancerous tissues were collected from surgery before other treatments were initiated. The adjacent paracancerous tissues were 5 cm away from the margins of the tumor. In addition, 30 pure chronic gastritis patients (disease control group) and 30 healthy subjects (healthy control group) who underwent physical examination were also enrolled in the same period. The average age of chronic gastritis patients and healthy subjects were 59.5 and 61.2 years, respectively. This study was performed in accordance with the rules of the Declaration of Helsinki of 1975 (revised in 2013). The study was approved by the institutional ethics committee of the First Affiliated Hospital of Henan University (No.2019053), and all subjects signed informed consent.

### Exosome Isolation

Preoperative fasting venous blood (4 ml) was collected using EDTA-anticoagulant tube and centrifuged at 3,000 g for 10 min within 2 h, then the plasma was further centrifuged at 12,000 g for 10 min at 4°C to remove cell debris. In addition, the second blood sample was collected 10 days after operation in twenty of the thirty patients who received surgical treatment. The processing method was the same as above. Subsequently, exosomes were isolated using ExoQuick solution (System Biosciences, United States) following the manufacturer’s instructions.

### Exosome Identification

The morphological characteristics of the purified exosomes were observed through transmission electron microscopy (TEM) and nanoparticle tracking analysis (NTA) as described previously [12]. Briefly, 10 μl of exosome suspension were absorbed onto carbon-coated cooper grids (200 mesh) for 1 min. Then the samples were washed with double-distilled water and negatively stained with 2% uranyl acetate solution for 1 min. Grids were visualized at 87000x in a Phillips Tecnai transmission electron microscope at 80 kV. The concentration and size distribution of the exosomes were determined using nanoparticle tracking analysis (NTA). Exosomes were diluted with PBS (1:1000) and then injected into the Zeta Potential/Particle Sizer NICOMPTM 380 ZLS analyzer (Santa Barbara, CA, United States). Particles were automatically tracked and sized based on brownian motion and the diffusion coefficient.

### Circular RNA Microarray Analysis

Total RNA of cancer cells derived exosome was isolated through exoRNeasy Midi Kit (Qiagen) and then used for circRNA microarray assay. The RNA quality was evaluated by capillary electrophoresis on Agilent 2100 Bioanalyzer (Agilent Technologies, CA, United States). The circRNA microarray assay was conducted *via* the Agilent human circRNA V6 Microarray by Shanghai Biotechnology Corporation. The microarray data have been uploaded to the GEO database (GSE202538).

### RNA Extraction and Quantitative Real-Time PCR

RNA extraction and qRT-PCR analysis were performed as our previous description [12]. Briefly, the total RNA from tissues was extracted using TRIzol reagent (Invitrogen), and exosomal RNA was extracted using a miRNeasy Micro Kit (QIAGEN) according to the manufacturers’ instructions. The quantity and quality of the RNA were evaluated using a NanoDrop spectrophotometer (Thermo Fisher Scientific). Five top upregulated circRNAs (hsa_circ_0028855, hsa_circ_0086471, hsa_circ_0049058, hsa_circ_0021091 and hsa_circ_0015286) were selected as candidate molecules for preliminary clinical validation. The primer sequences are shown in Supplementary Table S1. The relative expression levels of the target circRNA was calculated using the 2^−ΔΔCT^ method. All results are expressed as the mean ± SD of three independent experiments.

### Serum Tumor Marker Analysis

The traditional serum tumor markers CEA and CA19-9 were measured on Roche COBAS E602 Immunology Analyzer (Roche Diagnostics, China). The reference intervals of CEA and CA19-9 are 0–5.0 ng/ml and 0–35.0 U/ml respectively.

### Statistical Analysis

SPSS 20.0 and GraphPad Prism 7.0 Software were used for all of the statistical analyses. Comparisons of hsa_circ_0015286 expression levels among different groups were analyzed using a Student’s *t*-test or one-way ANOVA. ROC curve and Kaplan-Meier survival plot were established to evaluate diagnostic and prognostic capacity of exosomal hsa_circ_0015286. The relationships between hsa_circ_0015286 expression levels and clinicopathological parameters was further analyzed by t-tests. *p* < 0.05 was considered as statistically significant.

## Results

### Screening of Cancer-Specific Exosomal circRNAs by Circular RNA Microarray Assay

**FIGURE 1:**
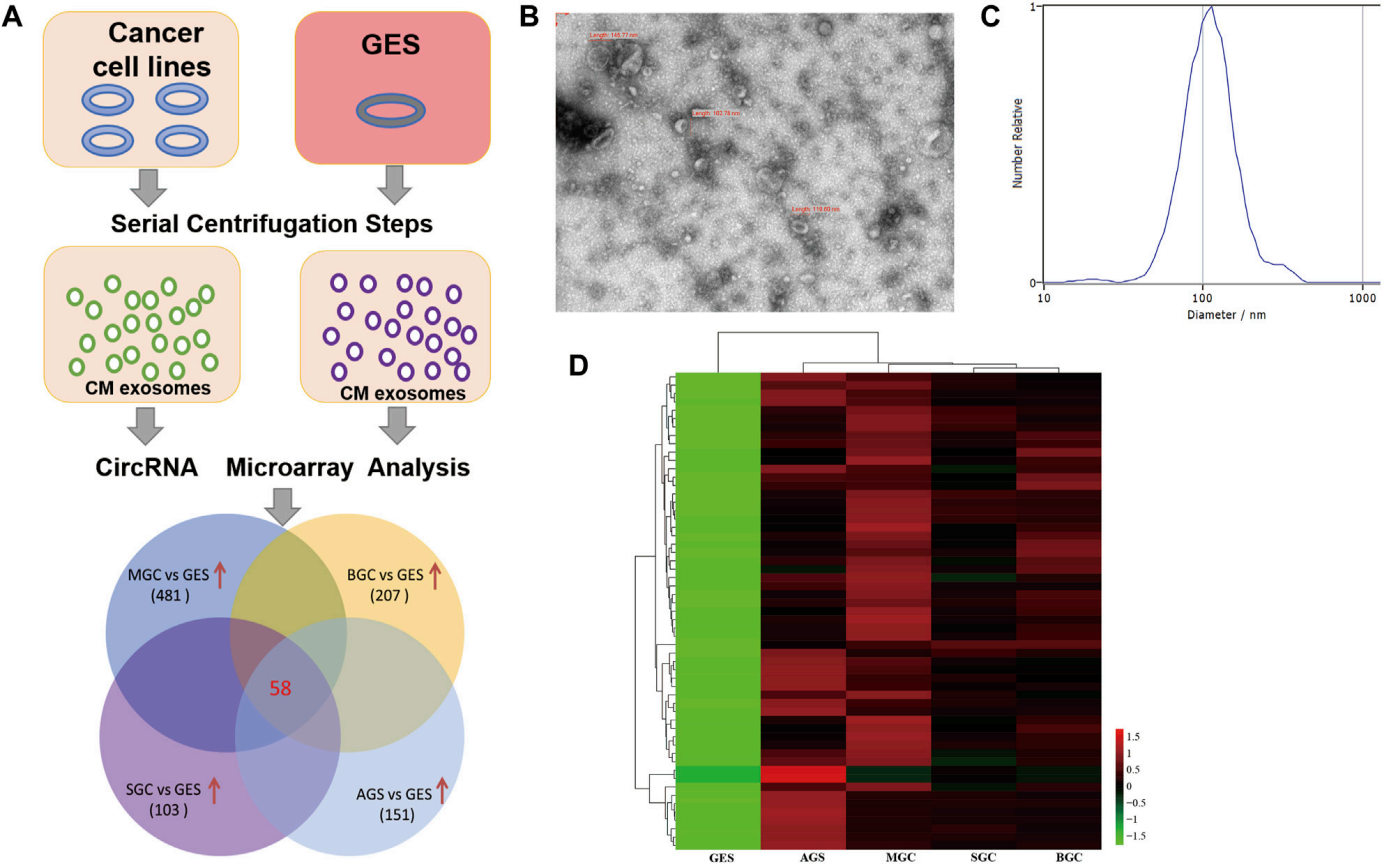
Screening of cancer-specific exosomal circRNAs through circRNA microarray assay. **(A)**: Flow diagram of the screening strategy; **(B)**: Representative transmission electron microscopy image of purified exosomes; **(C)**: NTA of the size distribution and number of exosomes; **(D)**: Heatmap result of upregulated circRNAs based on circRNA microarray analysis.

The screening strategy for identification of cancer-specific exosomal circRNAs is illustrated in Figure 1A. Culture media from different cancer cell lines (MGC803, BGC823, SGC7901, and AGS) and GES-1 were collected, then exosomal RNA was isolated and circRNA microarray assay was conducted to detect differential circRNA expression. Transmission electron microscopy (TEM) and nanoparticle tracking analysis (NTA) were utilized to characterize and quantify the isolated exosomes, a typical teacup-like structure was observed with a diameter of 80–120 nm (Figures 1B,C). Based on the microarray data, the Venn diagram showed that 58 circRNAs were highly expressed (fold change of ≥2 and *p* ≤ 0.05) in cancer cell derived exosomes compared with those in GES-1. The heatmap showed the different expression levels of the 58 upregulated circRNAs (Figure 1D).

### Exosomal hsa_circ_0015286 Expression Was Upregulated in Cancer Cells, GC Plasma and Tissues

**FIGURE 2:**
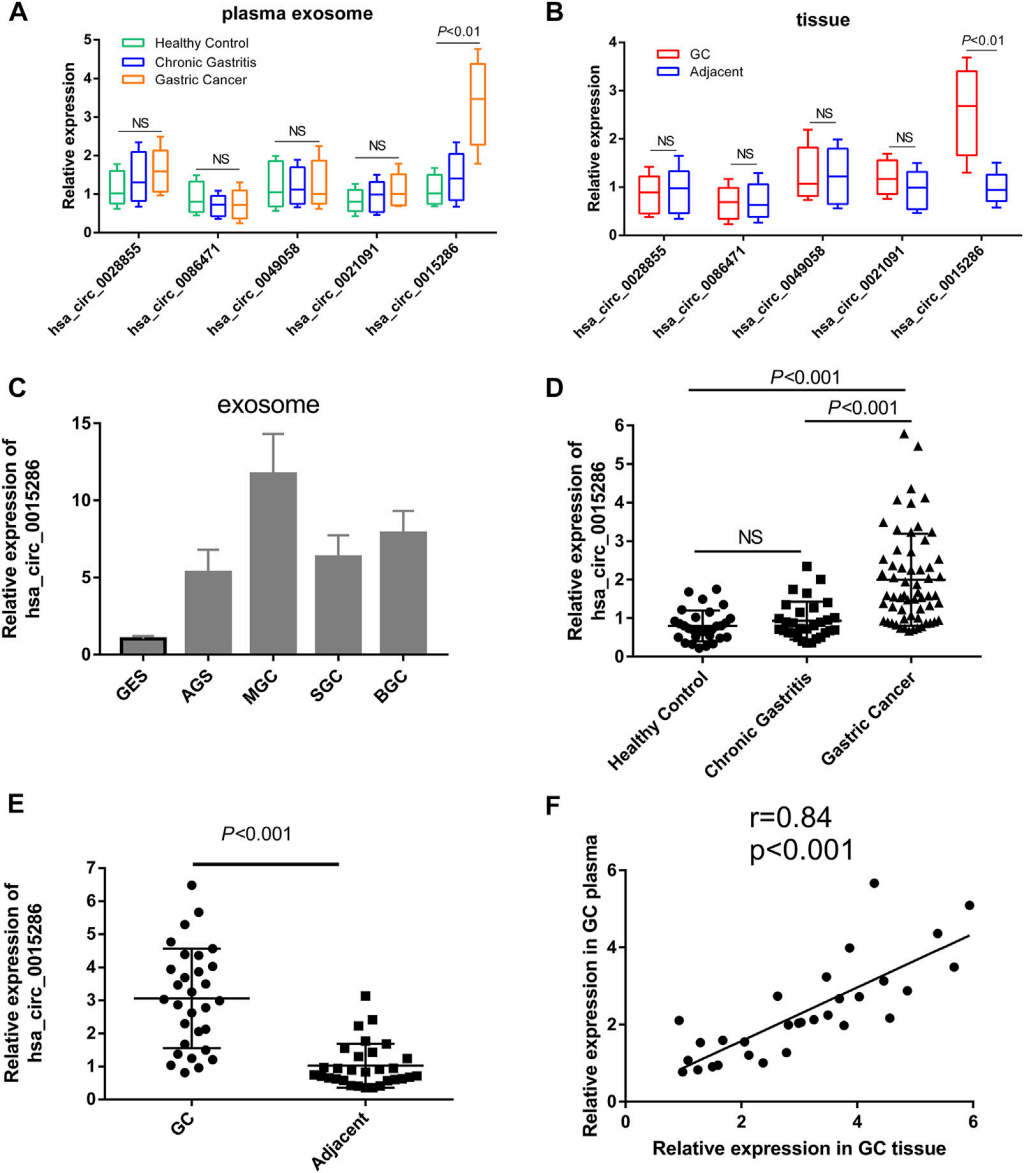
Expression levels of candidate circRNAs in cancer cells and clinical specimens. **(A)** The relative expression of five upregulated circRNAs in plasma exosomes of preliminary clinical subjects; **(B)** The relative expression of five upregulated circRNAs in tissue samples of preliminary clinical subjects; **(C)**: Hsa_circ_0015286 expression levels in exosomes derived from cancer cell lines and control GES cell; **(D)**: Hsa_circ_0015286 expression levels in plasma exosomes of GC patients, chronic gastritis patients and healthy control; **(E)**: Hsa_circ_0015286 expression levels in 30 paired GC tissues and adjacent normal tissues; **(F)**: The relationship of hsa_circ_0015286 expression levels in GC tissues and plasma samples.

Subsequently, in order to confirm the robustness of the circRNA microarray assay and investigate the clinical application value of candidate molecules, we detected the expression level of five top upregulated circRNAs using qRT-PCR in the plasma exosomes of 10 GC patients, 10 chronic gastritis patients and 10 healthy controls. Eight paired cancer tissue and paracancer tissue were also used for preliminary clinical validation. More interesting, besides the high expression in cancer cell derived exosomes, hsa_circ_0015286 was the uniquely upregulated circRNA in the plasma exosomes and tissues of GC patients compared with control groups. While there was no significant difference in the expression of four other molecules when validated with preliminary clinical specimens (Figures 2A–C). Thus, the following work was focused on this particular circRNA molecule. Sixty patients with newly diagnosed GC, 30 chronic gastritis patients (disease control group) and 30 healthy subjects (healthy control group) were enrolled for further clinical validation. The RT-PCR results showed that plasma exosomal hsa_circ_0015286 level was significantly upregulated in the GC group compared with chronic gastritis patients and healthy control (Figure 2D, *p* < 0.001).

Then, the expression of hsa_circ_0015286 were tested in 30 paired GC tissues and paracancerous tissues. The expression levels of hsa_circ_0015286 in GC tissue were also substantially higher than that in paracancerous tissue (Figure 2E, *p* < 0.001). We further analyzed their relationship with plasma expression by Spearman correlation analysis, a significant positive correlation was observed between hsa_circ_0015286 expression in GC tissues and plasma samples (Figure 2F).

### The Diagnostic Performance of Plasma Exosomal hsa_circ_0015286 in Gastric Cancer Patients

**FIGURE 3:**
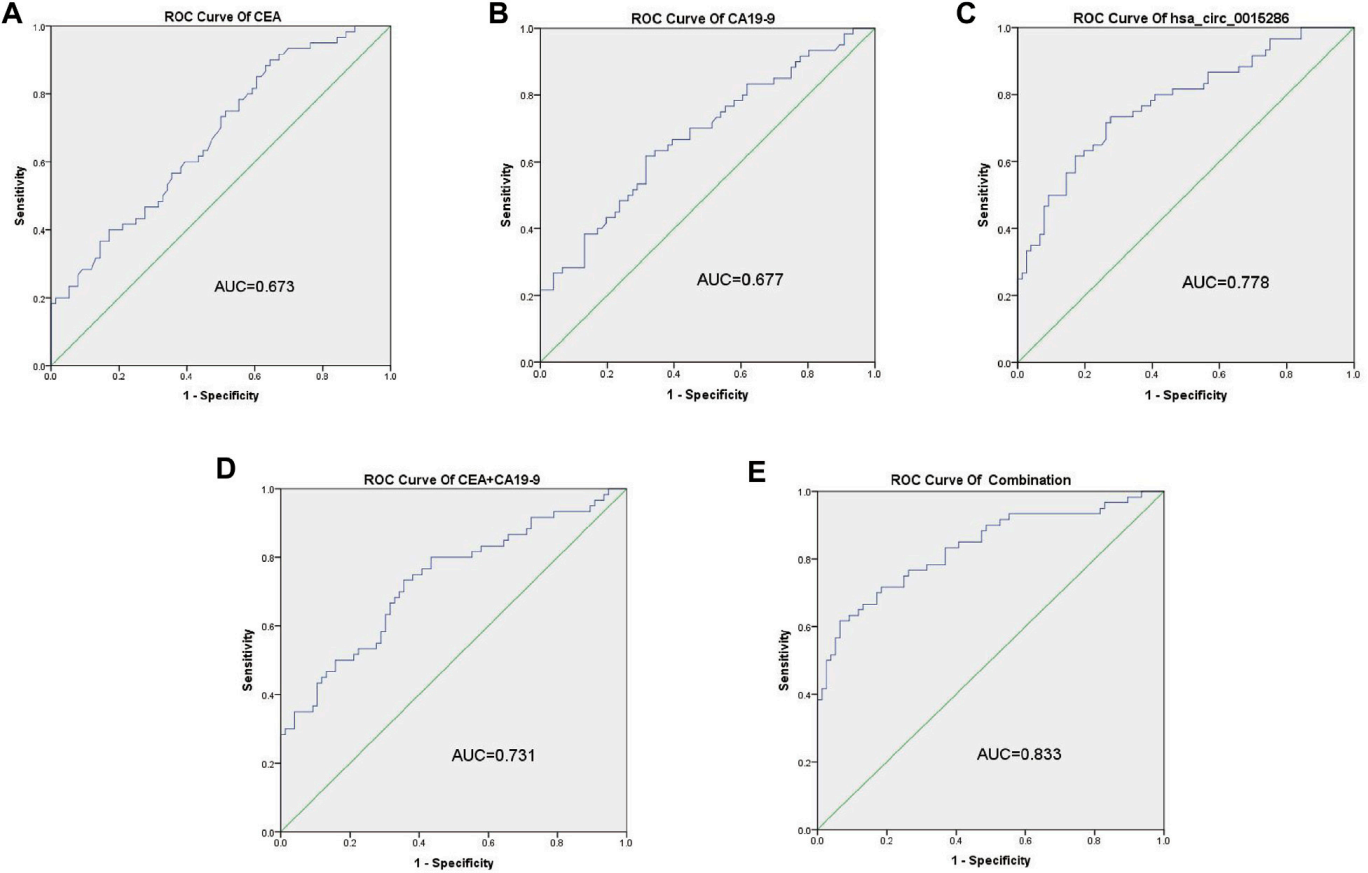
ROC curve analysis of hsa_circ_0015286, CEA and CA19-9. **(A–C)**: ROC curve analysis of serum CEA, CA19-9 and exosomal hsa_circ_0015286 in GC patients versus controls, respectively; **(D)**: ROC curve analysis of the combination of CEA and CA19-9; **(E)**: ROC curve analysis of the combination of CEA, CA19-9 and exosomal hsa_circ_0015286 in GC patients versus controls.

The potential diagnostic value of plasma exosomal hsa_circ_0015286 was evaluated through ROC curve, compared with the traditional tumor marker CEA and CA19-9. As shown in Figure 3 A, B and C, the AUC value of exosomal hsa_circ_0015286 was 0.778 (CI = 0.699–0.857, *p* < 0.001), which was better than serum CEA (AUC = 0.673, CI = 0.583–0.763, *p* < 0.01) and CA 19–9 (AUC = 0.677, CI = 0.556–0.738, *p* < 0.01). At the optimal cut-off value, the sensitivity and specificity were 0.821 and 0.657, respectively. What is more, the AUC value was up to 0.833 (CI = 0.762–0.904, *p* < 0.001) when combined with CEA and CA 19–9, which was better than that of pairwise combination (Figures 3D,E). These results showed that exosomal hsa_circ_0015286 may serve as an effective diagnostic marker for GC.

### Dynamic Monitoring and Potential Prognostic Value of Plasma Exosomal hsa_circ_0015286 in Gastric Cancer Patients

**FIGURE 4:**
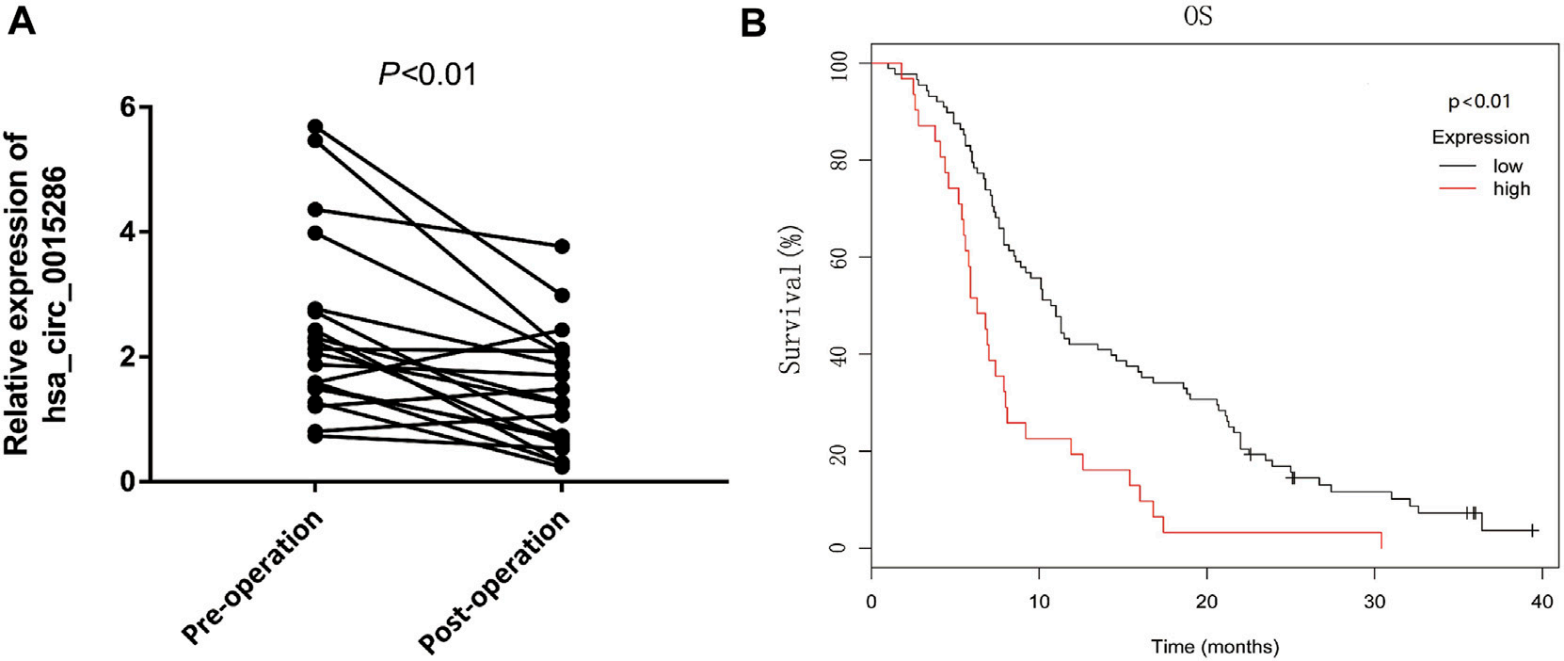
Dynamic monitoring and potential prognostic value of plasma exosomal hsa_circ_0015286 in GC patients. **(A)**: Hsa_circ_0015286 expression levels in 20 paired GC patients before and after surgery; **(B)**: Kaplan-Meier survival curves for clinical outcomes of GC patients with different level of exosomal hsa_circ_0015286.

To further investigate whether the expression levels of circulating hsa_circ_0015286 are associated with tumor burden, we compared the difference of exosomal hsa_circ_0015286 levels in 20 GC patients’ plasma before and after operation. As observed, the expression levels of exosomal hsa_circ_0015286 were remarkably downregulated 10 days post-surgery (*p* < 0.01, Figure 4A).

In addition, sixty GC patients were divided into high and low group based on the plasma expression levels of exosomal hsa_circ_0015286. Interestingly, the overall survival of GC patients in low expression group were longer than the high expression group (*p* < 0.01, Figure 4B). These results provided evidence that exosomal hsa_circ_0015286 may be used for dynamic monitoring and prognosis prediction.

### Correlation of Exosomal hsa_circ_0015286 With Clinicopathological Characteristics

The relationship of exosomal hsa_circ_0015286 expression levels with clinicopathological parameters of GC patients was summarized. As shown in Table 1, the expression levels of plasma exosomal hsa_circ_0015286 were closely related with tumor size (*p* < 0.001), T stage (*p* < 0.001) and lymph node metastasis (*p* < 0.001). Nevertheless, there was no correlation between its levels with age, gender, differentiation, CEA and CA19-9.

**TABLE 1:** The relationship of plasma exosomal hsa_circ_0015286 expression levels and clinicopathologic features of GC patients.

Characteristics	Number	Mean ± SD	*p* value
Gender			0.286
Male	32	3.24 ± 1.23	
Female	28	2.92 ± 1.05	
Age, years			0.159
<60	23	3.07 ± 1.85	
≥60	37	3.72 ± 1.63	
Tumor size, cm			<0.001^*^
<5	31	1.76 ± 0.68	
≥5	29	4.19 ± 1.59	
Differentiation			0.247
Poor + Moderate	40	2.88 ± 1.71	
Well	20	3.45 ± 1.92	
T stage			<0.001*
T1+T2	27	2.57 ± 0.96	
T3+T4	33	3.93 ± 1.42	
Lymph node metastasis			<0.001*
No	34	2.05 ± 1.14	
Yes	26	4.36 ± 1.55	
CEA, ng/ml			0.603
<5	35	3.04 ± 1.63	
≥5	25	3.25 ± 1.39	
CA19-9, U/ml			0.162
<35	38	2.79 ± 1.48	
≥35	22	3.37 ± 1.61	

*Indicate significance at *p* < 0.05.

CEA, carcinoembryonic antigen; CA19-9, Carbohydrate antigen19-9.

## Discussion

Early screening and diagnosis are pivotal to improve the 5-year survival rates of GC patients. However, traditional serum tumor biomarkers, such as CEA, CA 19–9, and CA 72–4, exhibit low sensitivity and specificity in GC diagnosis [13]. Meanwhile endoscopy detection, the gold standard for GC diagnosis, is invasive and uncomfortable for patients [14, 15]. As an important technique of liquid biopsy, accumulating evidence has demonstrated that tumor-specific proteins and RNA in exosomes exhibit an enormous potential in cancer diagnosis and prognosis judgment. Importantly, exosome detection has multiple advantages including non-invasiveness, convenient, rapid and higher sensitivity [16-19].

Li et al. first reported the existence and enrichment of circRNAs within cancer-derived exosomes *via* RNA-seq analysis [8, 9]. Moreover, previous studies showed that circRNAs are easy to be enriched in exosomes compared with linear RNA, and cancer-specific circRNAs can be transferred into the peripheral circulation *via* exosomes. Thus, the test of exosomal circRNA in peripheral blood as new biomarker may be feasible for tumor diagnosis [20, 21]. In this study, firstly cancer-specific exosomal circRNAs were identified by circRNA microarray assay. Next hsa_circ_0015286 was found that remarkably upregulated in GC tissue, plasma and cancer cells compared with normal controls. More importantly, a significant positive correlation was observed between its expression in GC tissues and plasma samples, which implying the preferable diagnostic and prognostic value for GC patients. ROC curve analysis demonstrated that exosomal hsa_circ_0015286 could be used as an independent biomarker for GC diagnosis or combined with CEA and CA19-9 to improve the diagnostic efficacy. Similar to our results, several aberrant expressions of exosomal circRNAs have been found and represent potential biomarkers and therapeutic targets in various types of cancer, including GC [22-24]. For instance, researchers found that hsa_circ_0065149 and hsa_circ_0000419 were remarkably decreased in plasma exosomes and tissues of GC patients, showing better performence for early GC diagnosis than conventional tumor biomarkers [25, 26]. In addition, circ-KIAA1244 released from GC tissues derived exosomes could serve as novel circulating biomarker for GC screening [27]. Of course, most of these reports including ours are preliminary and exploratory, more clinical studies are required to validate the diagnostic performance in future.

Besides the diagnostic application, the abnormal expression of circulating exosomal circRNAs have been proved to be associated with the clinicopathological characteristics of patients. For example, circ-IARS was reported highly expressed in serum exosomes of patients with metastatic pancreatic cancer and was associated with TNM stage, liver metastasis and tumor vascular invasion [28]. The higher expression of exosomal circ-RanGAP1 in GC was significantly related to lymph node metastasis, TNM stage, and poor survival [29]. In addition, exosomal circFECR1 was also found upregulated in serum of small cell lung cancer (SCLC) patients, its expression can reflect the clinical reaction to chemotherapy and prognostic evaluation [30]. Our findings demonstrated that the upregulation of exosomal hsa_circ_0015286 was also positively associated with tumor size, T stage and lymph node metastasis. Moreover, the expression level of exosomal hsa_circ_0015286 dramatically reduced 10 days post-surgery. The overall survival of patients with low hsa_circ_0015286 expression was longer than those with high expression in Kaplan-Meier survival plot analysis. These indicate that exosomal hsa_circ_0015286 might be a useful prognostic biomarker and involve in the progression of GC. Next, it is necessary to carry out large-scale clinical verification, long-term follow-up and functional analysis to further confirm our conclusions.

In summary, our study indicates that exosomal hsa_circ_0015286 is highly expressed in GC patients and might be a promising noninvasive biomarker for the diagnosis and prognosis evaluation of GC.

## Data Availability

The raw data supporting the conclusion of this article will be made available by the authors, without undue reservation.
