# Supplementary material for: Plasma Exosomal hsa_circ_0015286 as a Potential Diagnostic and Prognostic Biomarker for Gastric Cancer
Source: Pathol Oncol Res. 2022 Jun 9;28:1610446. doi: 10.3389/pore.2022.1610446 (PMC9218071; doi:10.3389/pore.2022.1610446)
Supplement: Supplementary file 1 [file Table1.docx]

**Table S1:** Sequences of primers used for qRT-PCR in this study

| **Primers** | **Forward** | **Reverse** |
| --- | --- | --- |
| GAPDH | 5´-CTCTGCTCCTCCTGTTCGAC-3´ | 5´-GCGCCCAATACGACCAAATC-3´ |
| hsa_circ_0028855 | 5'- CCCACTTGCTGAAAAGGTGC-3' | 5'- AAAAGGAGGTCTTCTCGGGC-3' |
| hsa_circ_0086471 | 5'- GCGAGCTTCTACTTCTAAGTCT-3' | 5'- TTAGCCTCCAGAGTCTGATCTT-3' |
| hsa_circ_0049058 | 5' - CCGCCACACGTAACTGAGAT-3' | 5' - AAGTGGTCGAACCCGACATC-3' |
| hsa_circ_0021091 | 5'- ATGTAACTGACCACCCAGGC-3′ | 5'- TCAAGGTTGACAGTTGGGCA-3' |
| hsa_circ_0015286 | 5´-ATCATCGCGGTCAAAGTAGTA-3´ | 5´- ATGCAATGGAGTGTGCATTAC-3´ |
